# Changes in Immunogenicity during the Development of Urinary Bladder Cancer: A Preliminary Study

**DOI:** 10.3390/ijms17030285

**Published:** 2016-02-25

**Authors:** Wojciech Jóźwicki, Anna A. Brożyna, Jerzy Siekiera, Andrzej T. Slominski

**Affiliations:** 1 Department of Tumour Pathology and Pathomorphology, Faculty of Health Sciences, Nicolaus Copernicus University Collegium Medicum in Bydgoszcz, Romanowska Street 2, Bydgoszcz 85-796, Poland; anna.brozyna@cm.umk.pl; 2 Department of Tumour Pathology and Pathomorphology, Oncology Centre-Prof. Franciszek Łukaszczyk Memorial Hospital, Romanowska Street 2, Bydgoszcz 85-796, Poland; 3 Department of Urology, Oncology Centre-Prof. Franciszek Łukaszczyk Memorial Hospital, Romanowska Street 2, Bydgoszcz 85-796, Poland; siekieraj@co.bydgoszcz.pl; 4 Departments of Dermatology and Pathology, University of Alabama at Birmingham, Birmingham, AL 35294, USA; aslominski@uabmc.edu; 5 Laboratory Service of the VA Medical Center, 700 South 19th Street, Birmingham, AL 35233, USA

**Keywords:** urinary bladder cancer, tumor-infiltrating lymphocytes (TILs), non-classic differentiation number (NDN), tissue invasion type, Tregs

## Abstract

In the present study, we evaluated tumor-infiltrating lymphocytes (TILs) and blood regulatory T lymphocyte (Tregs, CD4+/CD25+/FoxP3+) expression in bladder cancer patients. The number of CD4+, CD8+, CD25+, FoxP3+ and CD20+ TILs was analyzed in association with clinico-pathomorphological features. In more advanced metastasizing tumors, showing non-classic differentiation (ND) and a more aggressive tissue invasion type (TIT), the number of TILs decreased. A low number of CD4+ TILs was associated with poor prognosis. Similarly, Treg frequency before surgery and after surgical treatment was significantly lower in more advanced tumors. The changes in TILs, as well as of local and systemic Tregs, were accompanied by changes in the histological phenotype of urothelial carcinoma regarding pT stage, NDs, TIT, and clinical outcomes. The number of TILs and the frequency of blood Tregs (indicators of antitumor response) may be essential for choosing an immunotherapy that is adjusted to the immune status according to the phase of tumor growth. Moreover, a significant reduction in the number of CD4+ and CD8+ TILs with the development of NDs in more advanced tumors may be associated with lower tumor immunogenicity, resulting in immune tolerance towards tumor tissue. These observations and the tendency of urothelial bladder carcinoma to undergo NDs in a heterogeneous manner during tumor progression suggest complex interactions between bladder cancer immunogenicity and stages of tumor progression.

## 1. Introduction

The prognosis of urothelial carcinoma of the urinary bladder is determined by the tumor stage and its pathological features ([1,2,3], reviewed in [4]). Defining histological and clinical indicators of malignancy enabled better characterization of the neoplastic progression in bladder cancer. The prognosis of patients with pT2–pT4 tumors is significantly less favorable compared to pT1 tumors [2,3,5]. Specifically, tumors at pT2–pT4 exhibit a more aggressive tissue invasion type (TIT) compared with pT1 tumors), which are limited to the mucosa and the submucosa of the urinary bladder wall. Moreover, deep-infiltrating tumors (pT2–pT4) show a tendency toward multidirectional differentiation, which is connected with the development of the ability to metastasize [1,2,3,6]. Our previous research has indicated that histological and clinical markers of urothelial carcinoma of the bladder correlate with molecular events suggesting a complex pathomechanism of bladder cancer malignancy [5,6,7]. That study showed higher representation of tumor OCT4+ cells (the stem cell phenotype) was associated with tumor progression defined as invasion of mucosa (the first stage), infiltration of the muscle layer of the bladder wall (the second stage), and metastatic process (the third stage) [7]. Similarly, expression of stem cell markers was related to poor prognosis in other tumors [8,9,10]. We have also shown that RCAS1 (receptor-binding cancer-associated surface antigen) expression in neoplastic cells or in tumor microenvironment cells indicates the tumor escape from immune surveillance during malignant progression [11]. Similarly, in patients with ovarian cancer who did not respond to therapy, a significantly higher expression of RCAS1 within both the cancer cells and microenvironmental TAMs (tumor-associated macrophages) or CAFs (cancer associated fibroblasts) was observed in comparison to patients who responded to the treatment [12].

There is a shortage of information on the changes in tumor immunogenicity during the development and progression of urinary bladder cancer, especially in relation to clinical and pathomorphological features. Therefore, the aim of this study is to evaluate a correlation between presence of CD4+, CD8+, CD20+, CD25+ and FoxP3+ tumor-infiltrating lymphocytes (TILs), and clinico-pathological features of bladder cancer, as well as to assess blood Treg (*i.e.*, CD4+CD25+FoxP3+) frequency before (Treg-pre) and after (Treg-post) tumor excision in the early (pT1–pT2) and late (pT3–pT4) phases of tumor development. We have expected that changes of these variables in combination with selected pathomorphological features may be a predictive of changes in urothelial bladder cancer immunogenicity.

## 2. Results

### 2.1. Staging and Tumor-Infiltrating Lymphocytes (TILs) in Urinary Bladder Cancer

**Figure 1:**
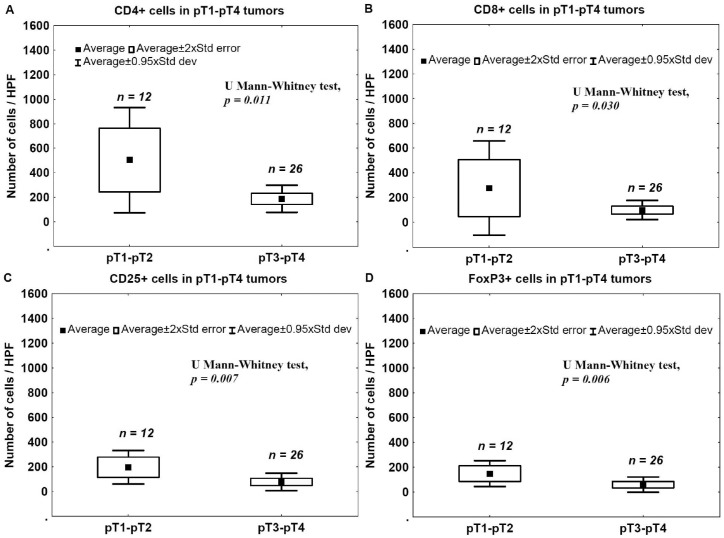

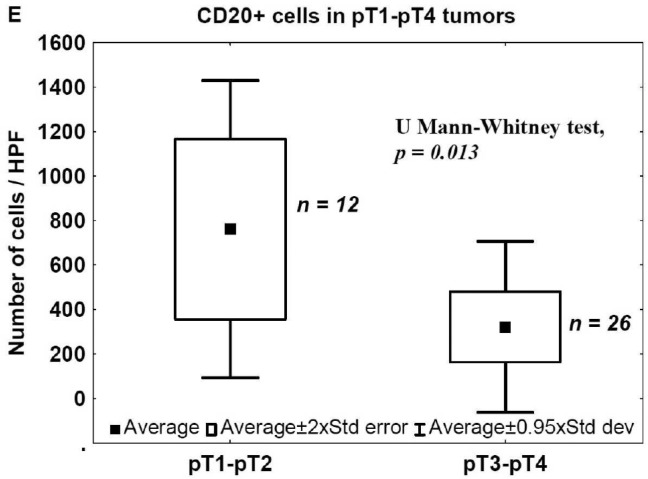
TILs in bladder cancers. Mean number of CD4+ (**A**); CD8+ (**B**); CD25+ (**C**); FoxP3+ (**D**) and CD20+ (**E**) infiltrating lymphocytes in pT1–pT2 *vs.* pT3–pT4 tumors.

To evaluate correlations between the advancement stage and the immunogenicity status of tumors, the number of CD4+, CD8+, CD25+, FoxP3+ and CD20+ TILs in invasive tumors was analyzed in relation to the phase of tumor development, as indicated by pT stage. The number of CD4+, CD8+, CD25+, FoxP3+ and CD20+ TILs in the late phase of tumor development (pT3–pT4) was significantly lower compared to that observed for less advanced tumors (pT1–pT2) (Figure 1A–E).

**Figure 2:**
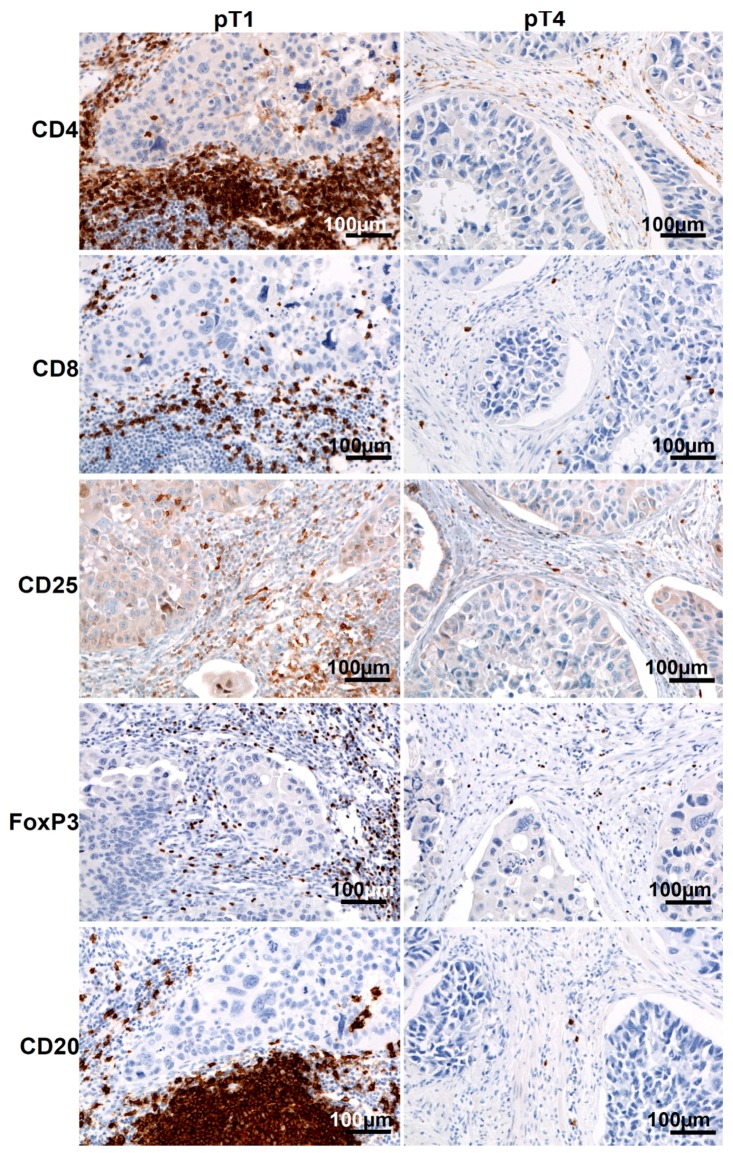
TILs in bladder cancers. Representative immunostaining of CD4, CD8, CD25, FoxP3 and CD20 TILs in pT1 (**left**) and pT4 (**right**) bladder cancers.

Representative immunostaining of CD4, CD8, CD25, FoxP3, and CD20 TILs in pT1 and pT4 cancers is presented in Figure 2.

### 2.2. Staging and Blood Tregs

**Figure 3:**
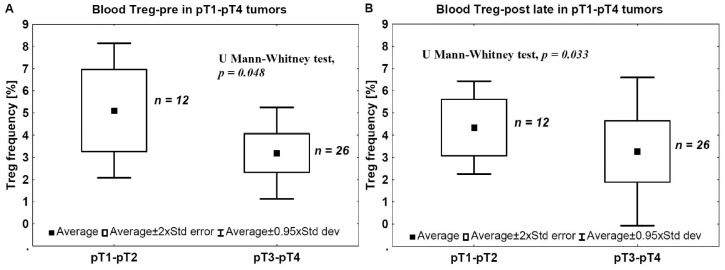
Tregs in bladder cancers. The mean frequency of blood Tregs before ((**A**) Treg-pre) and 7–10 days after ((**B**) Treg-post late) tumor excision was significantly lower in more advanced (pT2–pT3) than in pT1–pT2 tumors.

To evaluate the correlation between Tregs in the blood and the tumor advancement stage, the ratios of CD4+/CD25+/FoxP3+ lymphocytes were analyzed in the blood of patients with invasive tumors in relation to the phase of tumor development, as indicated by pT stage. In all cases, the blood Treg-pre (isolated before surgery) and Treg-post late (isolated 7–10 days after surgical treatment) frequencies were significantly lower in more advanced (pT3–pT4) than in less advanced (pT1–pT2) tumors (Figure 3A,B).

### 2.3. Nonclassic Differentiation Number (NDN) and TILs

**Figure 4:**
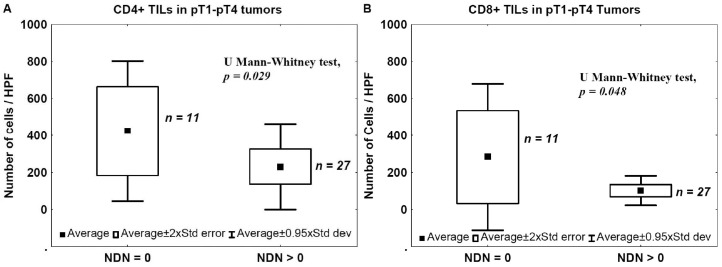
TILs and NDN. Mean number of CD4+ (**A**) and CD8+ (**B**) tumor-infiltrating lymphocytes in relation to tumor ability to develop non-classic differentiation. NDN, non-classic differentiation number.

The number of TILs was also analyzed in relation to NDN in pT1–pT4 tumors to evaluate the relationship between the NDN and tumor immunogenicity. The mean number of CD4+ TILs and CD8+ TILs was significantly lower in tumors that developed non-classic differentiation (NDN > 0) compared with samples from cancers with a classic histological pattern (NDN = 0) (Figure 4).

There was no significant correlation between NDN and the presence of CD20+, CD25+ and FoxP3+ TILs (data not shown).

### 2.4. Tumor Invasion Type (TIT) and TILs

**Figure 5:**
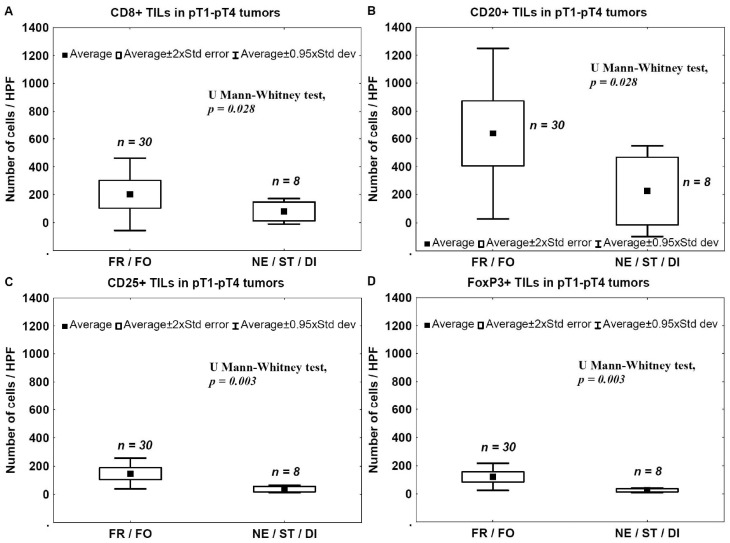
TILs and TIT. Mean number of CD8+ (**A**); CD20+ (**B**); CD25+ (**C**); and FoxP3+ (**D**) infiltrating lymphocytes in tumor samples in relation to the aggressiveness of tissue invasion. FR, frontal; FO, focal; NE, nested; ST, styloid; DI, dispersive.

The number of TILs was analyzed in relation to the type of tumor invasion to assess the relationship between tumor immunogenicity and the aggressiveness of cancer invasion. The number of TILs was significantly lower in tumors that had more aggressive type of invasion (nested, styloid, or dispersive; NE/ST/DI) compared to that observed in tumors with less aggressive type of invasion (frontal or focal; FR/FO) (Figure 5).

There was no significant correlation between TIT and the presence of CD4+ TILs (data not shown).

### 2.5. Metastasis and TILs

**Figure 6:**
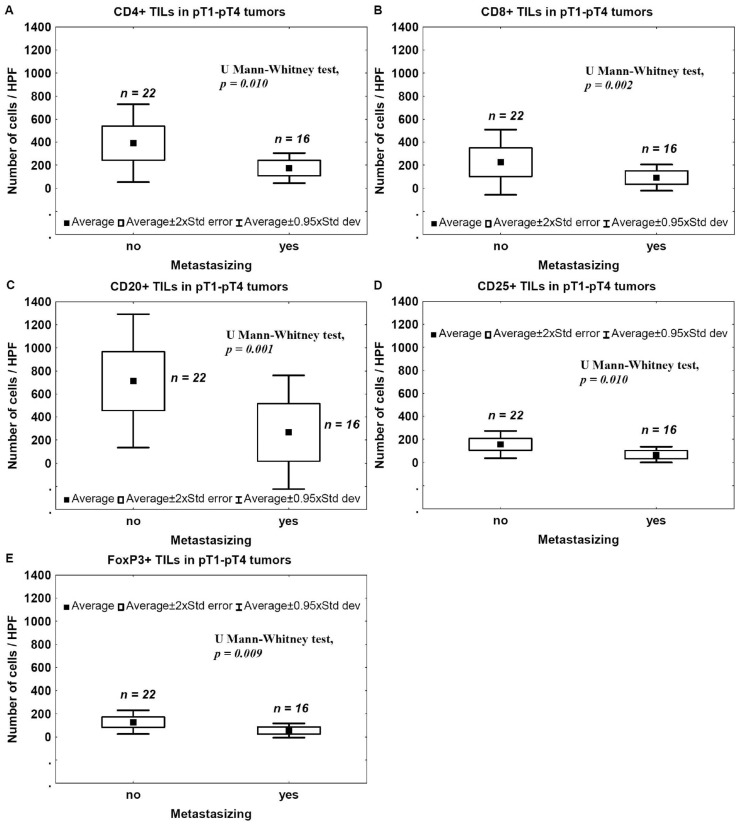
TILs and metastasis. Mean number of CD4+ (**A**); CD8+ (**B**); CD20+ (**C**); CD25+ (**D**) and FoxP3+ (**E**) TILs within invasive bladder cancer in relation to tumor ability to metastasize.

To assess a correlation between the presence of TILs and the metastatic potential, the number of CD4+, CD8+, CD25+, FoxP3+, and CD20+ TILs in non-metastasizing and metastasizing tumors was evaluated. Number of TILs expressing CD4, CD8, CD20, CD25, or FoxP3 was significantly lower in metastasizing tumors compared to non-metastasizing ones (Figure 6).

### 2.6. Survival and TILs

**Figure 7:**
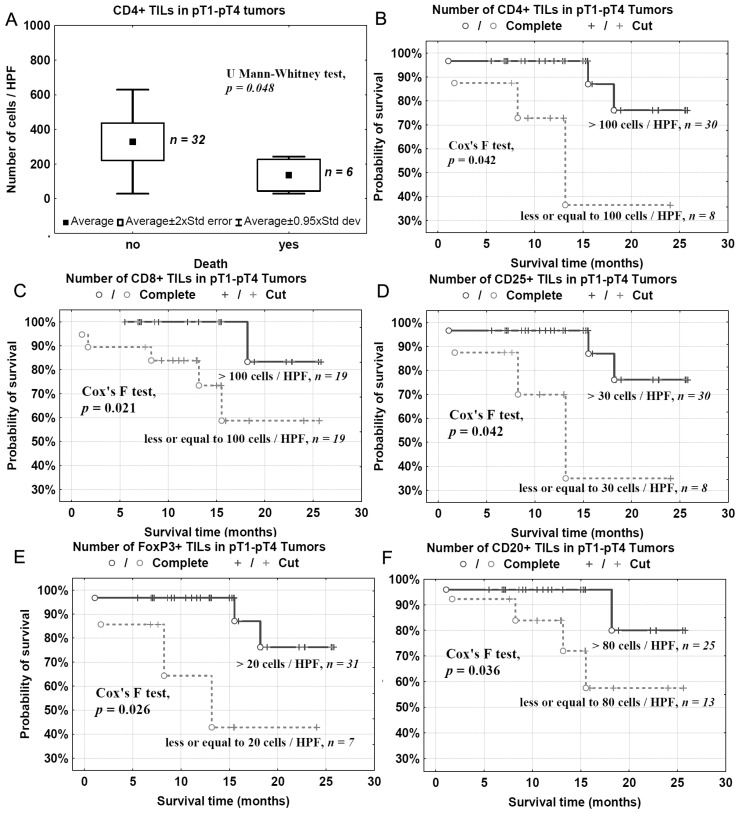
Mean number of CD4+ TILs in relation to patient death (**A**) and overall survival in relation to number of analyzed TILs/HPF: CD4+ (**B**); CD8+ (**C**); CD25+ (**D**); FoxP3+ (**E**) and CD20+ (**F**).

To evaluate the effects of TILs on the clinical outcome of patients with bladder cancer, CD4+, CD8+, CD25+, FoxP3+ and CD20+ TILs were analyzed in relation to patients’ death/survival. The mean number of CD4+ TILs was significantly lower in tumor samples from patients who had died than in tumor samples from surviving patients (Figure 7A). There was no significant correlation between patient death and the presence of CD8+, CD20+, CD25+ and FoxP3+ TILs (not shown). However the detailed analysis using the Kaplan-Meier method with experimentally-determined cut-off points showed that the lower number of analyzed TILs (CD4+, CD8+, CD20+, CD25+ and FoxP3+) was related to shorter overall survival (Figure 7B–F).

## 3. Discussion

In the present study, we analyzed a correlation between CD4+, CD8+, CD25+, FoxP3+, and CD20+ TILs and pathomorphological features (pT advancement, NDN, and the type of tissue invasion) as well as clinical outcomes (metastasis and patients’ death). We also measured Treg levels in the blood before and after tumor excision in two cohorts of patients with pT1–pT2 and pT3–pT4 tumors. We found that a low number of CD4+ TILs was associated with a poor prognosis (Figure 7). A detailed analysis also revealed that the levels of effector T cells, CD4+ and CD8+, were significantly lower in the late phase of tumor development (pT3–pT4) (Figure 1A,B) and in bladder cancers that developed non-classic differentiation (NDN > 0) (Figure 4). Furthermore, more advanced (pT3–pT4) tumors were characterized by a lower number of CD20+ B-cell TILs (Figure 1E) and TILs with a Treg phenotype, CD25+ and FoxP3+ (Figure 1C,D). Similarly, lower blood concentrations of Treg-pre and Treg-post were observed in more advanced cancers (Figure 3). Moreover, CD8+, CD20+, CD25+, and FoxP3+ TILs were seen less frequently in aggressive invasive tumors (NE/ST/DI) (Figure 5). Correspondingly, a reduced number of CD4+, CD8+, CD20+, CD25+, and FoxP3+ TILs was found in metastasizing tumors (Figure 6). The lower number of TILs detected in tumors with a more aggressive type of invasion in the present study was probably not associated with lower tumor immunogenicity. It might rather indicate a suppression of the antitumor response by systemic (blood) Treg activity. However, the observation of the lower frequency of blood Tregs in the late phase of tumor development requires additional analysis of TIL representation to better understand relationship between tumor phenotype and immune activity.

The differences in the presence of CD4+, CD8+, and CD20+ TILs in pT1–pT2 *vs.* pT3–pT4 tumors suggest that the immune response depends on the stage of tumor development, being less effective in more advanced tumors. Other authors have reported tumor-induced immune suppression and the immunological tolerance to tumor antigens as important elements limiting the effectiveness of the antitumor immune response [13]. We observed a lower representation of CD25+ and FoxP3+ TILs in advanced cancers and a reduction of blood Treg frequency after the excision of pT3–pT4 tumors. This suggests the presence of a local tumor-specific immune tolerance accompanied by a lower frequency of systemic (blood) and local Tregs. Thus, the immune suppression implemented by Tregs may be less important in the late phase of bladder cancer growth. The significantly-reduced number of CD25+ cells in more advanced tumors could reflect the lower antitumor response, related to less abundant regulatory T lymphocytes (FoxP3+). The lower number of TILs (CD4+, CD8+, CD25+, FoxP3+ and CD20+) observed in more advanced pT3–pT4 tumors compared to pT1–pT2 tumors (Figure 1 and Figure 2) may also reflect the lower immunogenicity of advanced lesions and the development of immune tolerance rather than the progression of biological malignancy with the appearance of new invasive cells. This may give physicians the opportunity to choose an immunotherapy that is adjusted to the immune status according to the phase of tumor growth. We also observed the lowering of number of CD25+ and FoxP3+ cells which could reflect the development of immune tolerance, resulting in more aggressive behavior, e.g., a more aggressive tissue invasion type (nested/styloid/dispersive) and metastasizing tumors. Thus, more aggressive tumor behavior is not only the result of the biological malignancy, but is also secondary to a decrease in antitumor response of the immune system and the development of immune tolerance to tumor antigens. In addition, we also found that increased NDN determined a higher risk of metastasizing and worse prognosis in more advanced deep-infiltrating tumors [5,6]. Other authors have suggested that in the neoplastic process, regulatory mechanisms of the immune system “are probably influenced by specific type of cancer, with its own unique set of genetic, epigenetic and inflammatory changes” and develop according to tumor advancement (reviewed in [14]). Therefore, it is possible that the decreasing tumor immunogenicity observed in more advanced bladder cancers is associated with the development of non-classic differentiation, especially during invasion of the muscle layer of the bladder wall [5,6]. This hypothesis is supported by our observation that the number of TILs is significantly lower in tumors that developed non-classic differentiation (NDN > 0). According to other reports, the immune system develops an antitumor response or immune tolerance, depending on a unique scenario regarding the amount of tumor antigens and the way they are presented [15,16,17,18]. We suggest that this scenario may be expanded with the histological diversity of bladder cancer that occurs during the development of non-classic differentiations (NDN > 0), which induce immunological tolerance toward differentiating phenotypically elements specific for urothelial cancer (squamous-like, glandular-like, clear cell-like, giant cell-like, plasmocytoid, lymphoma-like, sarcomatoid, and other) [1,5]. Therefore, we suggest that therapeutic strategies based on the regulation of immune-system activity should take into account the phase of bladder cancer development, as specified by several histoclinical characteristics. Therapies that inhibit the suppression of the immune system using anti-Treg drugs should be considered in bladder cancers when the frequencies of local and blood Tregs are highest and are accompanied by histoclinical phenomena such as low tumor advancement (pT1–pT2), classical differentiation (NDN = 0), a less aggressive type of invasion (FR/FO), lack of metastases (pN = 0), and a higher chance of survival. Accordingly, the initial treatment modality in bladder cancers with a lower number of TILs and a higher tumor advancement stage (pT3–pT4) showing non-classical differentiations (NDN > 0) with an aggressive type of invasion (NE/ST/DI), presence metastases (pN > 0), and with a higher risk of death, should be aimed at interrupting the immune tolerance to the tumor antigens, because the immune system has the potential to destroy any tumor cell if it is immunogenic [18,19]. We suggest that a better understanding of the mechanisms underlying the immune tolerance to bladder cancer warrants further targeted exploration of the non-classic differentiation phenomena.

## 4. Materials and Methods

### 4.1. Patients

Thirty-eight patients with urothelial bladder cancer who underwent cystectomy (or cystoprostatectomy) were included in this study. The mean age of the pT1–pT2 and pT3–pT4 cohorts was 62 and 63 years, respectively. The characteristics of the patients included in this study are presented in Table 1. The mean follow-up time of patients included into this study was 13.18 months (median 12.95 months, from 1.10 to 25.80 months).

Pathological assessment (p) of primary tumors advancement (T) was performed according to the World Health Organization (WHO) Classification of Malignant Tumors, as previously described [20]. The study was approved by the Committee of Ethics of Scientific Research of the Collegium Medicum of Nicolaus Copernicus University, Poland.

### 4.2. Immunohistochemistry

For immunohistochemical analysis, 4 μm-thick sections were cut from archival paraffin-embedded blocks and stained with anti-CD4, anti-CD8, anti-CD20, anti-CD25, and anti-FoxP3 antibodies, as summarized in Table 2. Before incubation with the primary antibodies (with the exception of anti-CD25), endogenous peroxidase was blocked with hydrogen peroxide for 5 min. Hematoxylin was used as a counterstain. Appropriate positive controls were included (see Table 2).

### 4.3. Immunohistochemistry Assessment

For each group of lymphocytes that infiltrated the tumor mass and/or localized in the vicinity of 2 HPF from the tumor, the mean number of CD4+, CD8+, CD20+, CD25+ and FoxP3+ cells within 20 HPF was evaluated.

### 4.4. Immunolabeling of Tregs in Peripheral Blood and Flow Cytometry

The Treg populations were analyzed before surgical removal (Treg-pre) and 7–10 days after the surgical treatment (Treg-post late). In the literature the different definitions of Tregs are used [21]. In present study we defined the Tregs as in our previous study [22] and as was proposed by other authors [23,24]. The samples that were used for the cytometric analysis of Treg populations in the whole blood of patients were prepared using the FoxP3 Staining Kit (Becton Dickinson, Franklin Lakes, NY, USA) according to the manufacturer’s instructions with some minor modifications. To 100 μL of blood, 20 μL of fluorescein isothiocyanate (FITC)-labeled CD4, 20 μL of allophycocyanin (APC)-labeled CD25 and 5 μL of allophycocyanin-cyanine 7 (APC-Cy7)-labeled CD45 were added. After 20 min of incubation with monoclonal antibodies (in the dark at room temperature (RT)), the cells were washed with stain buffer by centrifugation for 10 min at 250× *g*, fixed with 2 mL of freshly prepared Buffer A (for 10 min at RT), and centrifuged for 5 min at 500× *g*. Subsequently, the cells were washed with stain buffer (5 min at 500× *g*) and permeabilized with 0.5 mL of freshly prepared Buffer C (for 30 min at RT). After two additional washing steps (5 min at 500× *g*), the cells were stained with phycoerythrin (PE)-labeled anti-human FoxP3 antibody for 30 min (in the dark at RT), washed twice with stain buffer (5 min at 500× *g*), suspended in Cell Wash Buffer, and analyzed using a BD FACS Canto II flow cytometer and the BD FACS Diva software (Becton Dickinson). Samples were analyzed as described previously [22]. Briefly, for each sample, 3 × 10^4^ lymphocytes were collected and gated on an SSC × CD45 dot plot. Subsequently, the populations of CD4+/FITC, CD25+/APC, and double-positive CD4+/CD25+ cells were distinguished among the lymphocytes, and the gate of FoxP3+ cells was established on the CD4+/CD25^high^+ subpopulation, which are the only cells that exhibit a regulatory function in humans [23]. The frequency of Tregs was defined as the percentage of cells with the CD25^high^+/FoxP3+ phenotype in the subpopulation of CD4+ T lymphocytes.

### 4.5. Histological Assessment

Nonclassic differentiation number (NDN), which indicates a tendency for multidirectional differentiation, and tissue invasion type (TIT), which reflects the local tumor spread, were evaluated as described previously [7,11,20,25].

### 4.6. Statistical Analyses

The differences in TIL number in relation to the variables analyzed were assessed using the Mann–Whitney *U* test. The overall survival in relation to analyzed parameters (CD4+, CD8+, CD20+, CD25+ anf FoxP3+ TILs numbers) was analyzed using the Kaplan–Meier method. The statistical analyses were carried out using the STATISTICA data-analysis software (version 8.0; StatSoft, Inc., Tulusa, OK, USA). A *p* value < 0.05 was considered indicative of statistical significance.

## 5. Conclusions

We found that the number of CD4+, CD8+, CD25+, FoxP3+ and CD20+ TILs and the frequency of blood Tregs changed with the progression of the urinary bladder cancer. Changes in the representation of effector cells and local (TILs) and systemic (blood) Tregs were accompanied by changes in the histological phenotype of urothelial carcinoma in relation to pT stage, NDN, the type of tissue invasion, and clinical outcomes such as metastasis and patients’ death. The number of TILs and blood Tregs frequency (indicators of antitumor response) was significantly lower in more advanced tumors compared to less advanced ones. This observation may be essential for the assessment of the tumor immunological status, which would be taken into account when selecting an immunological treatment strategy. Moreover, this study indicates that the number of CD4+ and CD8+ TILs decreases with the development of new histological patterns, *i.e.*, non-classic differentiations in more advanced tumors (pT3–pT4), which can be associated with lower tumor immunogenicity, resulting in stronger immune tolerance to cancer. Taking into consideration that urothelial carcinoma of the bladder demonstrates a significant tendency toward the generation of different non-classic differentiations as the disease advances, the above factors represent a good target for further research aimed at better understanding of changing tumor immunogenicity during bladder cancer progression.

## Figures and Tables

**Table 1:** Clinico-pathomorphological characteristics of patients with urothelial bladder tumors.

Feature	Number
**pT1–pT2 cohort**	**12**
Gender	
F	1
M	11
pT	
pT1	4
pT2	8
NDN	
NDN 0	5
NDN 1	3
NDN 2	4
NDN ≥ 3	0
TIT	
FR/FO	12
NE/ST/DI	0
Metastasis	
No	10
Yes	2
Death	
No	12
Yes	0
**pT3–pT4 cohort**	**26**
Sex	
F	1
M	25
pT	
pT3	18
pT4	8
NDN	
NDN 0	6
NDN 1	8
NDN 2	8
NDN ≥ 3	4
TIT	
FR/FO	18
NE/ST/DI	8
Metastasis	
No	12
Yes	14
Death	
No	20
Yes	6

**Table 2:** Description of the antibodies used and their manufacturers, and of immunocytochemistry protocols and detection methods.

Antibody	Vendor	Epitope Retrieval	Primary Antibody (Dilution; Incubation Time)	Method of Detection	Positive Control
anti-CD4	Dako (Carpinteria, CA, USA)	PT-Link, High pH	RTU; 20 min	Mouse Linker/EnVision/DAB (Dako)	lymph node
anti-CD8	Dako	PT-Link, High pH	RTU; 20 min	EnVision/DAB (Dako)	lymph node
anti-CD20	Dako	PT-Link, High pH	RTU; 20 min	EnVision/DAB (Dako)	lymph node
anti-CD25	Ventana Medical Systems, Inc. (Tucson, AZ, USA)	manufacturer’s protocol	RTU; manufacturer’s protocol	UltraView Universal DAB (Ventana)	lymph node
anti-FoxP3	Abcam (Cambridge, UK)	PT-Link, Low pH (Dako)	1:100; O/N 4 °C	EnVision/DAB (Dako)	lymph node

O/N, overnight; DAB, 3,3′-diaminobenzidine; RTU, ready to use.

## Abbreviations

APC: allophycocyanin
CAFs: cancer associated fibroblasts
DI: dispersive
FITC: fluorescein isothiocyanate
FO: focal
FR: frontal
ND: nonclassic differentiation
NDN: nonclassic differentiation number
NE: nested
PE: phycoerythrin
RT: room temperature
ST: styloid
TAMs: tumor-associated macrophages
TIL: tumor-infiltrating lymphocyte
TIT: tissue invasion type
Treg: blood regulatory T lymphocyte

## References

[1] Lopez-Beltran A., Sauter G., Gasser T., Hartmann A., Schmitz-Dräger B.J., Helpap B., Ayala A.G., Tamboli P., Knowles M.A., Sidransky D., Eble J.N., Sauter G., Epstein J.I., Sesterhenn I.A. (2004). Tumours of the urinary system. Infiltrating urothelial carcinoma. WHO Classification of Tumours. Pathology and Genetics of Tumors of the Urinary System and Male Genital Organs.

[2] Madersbacher S., Hochreiter W., Burkhard F., Thalmann G.N., Danuser H., Markwalder R., Studer U.E. (2003). Radical cystectomy for bladder cancer today—A homogeneous series without neoadjuvant therapy. J. Clin. Oncol..

[3] Kaufman D.S., Shipley W.U., Feldman A.S. (2009). Bladder cancer. Lancet.

[4] Van den Bosch S., Witjes J.A. (2011). Long-term cancer-specific survival in patients with high-risk non-muscle-invasive bladder cancer and tumour progression: A systematic review. Eur. Urol..

[5] Jozwicki W., Domaniewski J., Skok Z., Wolski Z., Domanowska E., Jozwicka G. (2005). Usefulness of histologic homogeneity estimation of muscle-invasive urinary bladder cancer in an individual prognosis: A mapping study. Urology.

[6] Domanowska E., Jozwicki W., Domaniewski J., Golda R., Skok Z., Wisniewska H., Sujkowska R., Wolski Z., Jozwicka G. (2007). Muscle-invasive urothelial cell carcinoma of the human bladder: Multidirectional differentiation and ability to metastasize. Hum. Pathol..

[7] Jóźwicki W., Brożyna A.A., Siekiera J. (2014). Expression of OCT4a: The first step to the next stage of urothelial bladder cancer progression. Int. J. Mol. Sci..

[8] Thanan R., Pairojkul C., Pinlaor S., Khuntikeo N., Wongkham C., Sripa B., Ma N., Vaeteewoottacharn K., Furukawa A., Kobayashi H. (2013). Inflammation-related DNA damage and expression of CD133 and Oct3/4 in cholangiocarcinoma patients with poor prognosis. Free Radic. Biol. Med..

[9] Yin J.Y., Tang Q., Zhai L.L., Zhou L.Y., Qian J., Lin J., Wen X.M., Zhou J.D., Zhang Y.Y., Zhu X.W. (2015). High expression of OCT4 is frequent and may cause undesirable treatment outcomes in patients with acute myeloid leukemia. Tumour Biol..

[10] Li N., Deng W., Ma J., Wei B., Guo K., Shen W., Zhang Y., Luo S. (2015). Prognostic evaluation of Nanog, Oct4, Sox2, PCNA, Ki67 and E-cadherin expression in gastric cancer. Med. Oncol..

[11] Jóźwicki W., Brożyna A.A., Siekiera J., Slominski A.T. (2015). Expression of RCAS1 correlates with urothelial bladder cancer malignancy. Int. J. Mol. Sci..

[12] Jóźwicki W., Windorbska W., Brożyna A.A., Jochymski C., Basta P., Sikora J., Stasienko E., Dutsch-Wicherek M., Koper K., Wicherek L. (2011). The analysis of receptor-binding cancer antigen expressed on SiSo cells (RCAS1) immunoreactivity within the microenvironment of the ovarian cancer lesion relative to the applied therapeutic strategy. Cell Tissue Res..

[13] Escors D. (2014). Tumour immunogenicity, antigen presentation, and immunological barriers in cancer immunotherapy. New J. Sci..

[14] Blankenstein T., Coulie P.G., Gilboa E., Jaffee E.M. (2012). The determinants of tumour immunogenicity. Nat. Rev. Cancer.

[15] Pradeu T., Carosella E.D. (2006). On the definition of a criterion of immunogenicity. Proc. Natl. Acad. Sci. USA.

[16] Drake C.G., Jaffee E.M., Pardoll D.M. (2006). Mechanisms of immune evasion by tumors. Adv. Immunol..

[17] Laheru D.A., Jaffee E.M. (2005). Immunotherapy for pancreatic cancer—Science driving clinical practice. Nat. Rev. Cancer.

[18] Tran T., Burt D., Eapen L., Keller O.R. (2013). Spontaneous regression of metastatic melanoma after inoculation with tetanus-diphtheria-pertussis vaccine. Curr. Oncol..

[19] Dupage M., Mazumdar C., Schmidt L.M., Cheung A.F., Jacks T. (2012). Expression of tumour-specific antigens underlies cancer immunoediting. Nature.

[20] Jóźwicki W., Brożyna A.A., Siekiera J., Slominski A.T. (2015). Expression of vitamin D receptor (VDR) positively correlates with survival of urothelial bladder cancer patients. Int. J. Mol. Sci..

[21] Santegoets S.J., Dijkgraaf E.M., Battaglia A., Beckhove P., Britten C.M., Gallimore A., Godkin A., Gouttefangeas C., de Gruijl T.D., Koenen H.J. (2015). Monitoring regulatory T cells in clinical samples: Consensus on an essential marker set and gating strategy for regulatory T cell analysis by flow cytometry. Cancer Immunol. Immunother..

[22] Wicherek L., Jozwicki W., Windorbska W., Roszkowski K., Lukaszewska E., Wisniewski M., Brozyna A.A., Basta P., Skret-Magierlo J., Koper K. (2011). Analysis of Treg cell population alterations in the peripheral blood of patients treated surgically for ovarian cancer—A preliminary report. Am. J. Reprod. Immunol..

[23] Baecher-Allan C., Brown J.A., Freeman G.J., Hafler D.A. (2001). CD4+CD25^high^ regulatory cells in human peripheral blood. J. Immunol..

[24] Zhang D., Tu E., Kasagi S., Zanvit P., Chen Q., Chen W. (2015). Manipulating regulatory T cells: A promising strategy to treat autoimmunity. Immunotherapy.

[25] Jóźwicki W., Skok Z., Brożyna A., Siekiera J., Wolski Z., Domaniewski J. (2010). Prognostic and diagnostic implications of histological differentiation in invasive urothelial cell carcinoma of the bladder: Variant or non-classic differentiation number. Cent. Eur. J. Urol..

